# Use of Microbiota to Fight Mosquito-Borne Disease

**DOI:** 10.3389/fgene.2020.00196

**Published:** 2020-03-10

**Authors:** Wei Huang, Sibao Wang, Marcelo Jacobs-Lorena

**Affiliations:** ^1^Department of Molecular Microbiology and Immunology, Johns Hopkins Malaria Research Institute, Johns Hopkins Bloomberg School of Public Health, Baltimore, MD, United States; ^2^CAS Key Laboratory of Insect Developmental and Evolutionary Biology, Institute of Plant Physiology and Ecology, Shanghai Institutes for Biological Sciences, Chinese Academy of Sciences, Shanghai, China

**Keywords:** insect microbiota, arboviruses, malaria, paratransgenesis, mosquito-pathogen interactions

## Abstract

Mosquito-borne diseases cause more than 700 million people infected and one million people die (Caraballo and King, 2014). With the limitations of progress toward elimination imposed by insecticide- and drug-resistance, combined with the lack of vaccines, innovative strategies to fight mosquito-borne disease are urgently needed. In recent years, the use of mosquito microbiota has shown great potential for cutting down transmission of mosquito-borne pathogens. Here we review what is known about the mosquito microbiota and how this knowledge is being used to develop new ways to control mosquito-borne disease. We also discuss the challenges for the eventual release of genetically modified (GM) symbionts in the field.

## Introduction

Mosquito vectors mainly include three genera, *Anopheles*, *Aedes*, and *Culex*. Spread of disease is via the bite of infected female mosquitoes. The pathogens include malaria, dengue, Chikungunya, Zika, Yellow fever, and West Nile and they lead to more than one million deaths every year (WHO, 2016; Rosenberg et al., 2018). Presently, strategies to control mosquito-borne diseases are limited to mosquito population reduction and in case of malaria, to drugs. No drugs are available to treat viral diseases. With the current unavailability of a vaccine (with the exception of yellow fever) that protects from any of the mosquito-borne pathogens (Cheeseman et al., 2012; Ferguson, 2018) and with the widespread of insecticide resistance of mosquitoes (Ranson and Lissenden, 2016), new weapons to fight these diseases are urgently needed.

Insect microbiota are involved in many important biological processes such as nutrition, digestion, sexual reproduction, development, and refractoriness to pathogens (Douglas, 2014). Bacteria such as *Wolbachia*, can shorten the life span of some mosquito species (McMeniman et al., 2009) and block virus mosquito infection and dissemination (Moreira et al., 2009; van den Hurk et al., 2012). In recent years, increasing interest has been shown in employing symbiotic bacteria to control mosquito-borne diseases.

## Gut Microbiota Diversity and Distribution in Mosquitoes

The mosquito gut microbiota includes prokaryotes, viruses, and eukaryotic microbes. In this review, we focus on prokaryotes and eukaryotic microbes. The mosquito gut microbiota is mainly acquired from the environment (Wang et al., 2017; Strand, 2018), and its composition is highly dynamic, varying greatly with species, nutrition, stage of mosquito development, and geography (Tchioffo et al., 2015; Minard et al., 2017; Novakova et al., 2017; Bascunan et al., 2018; Krajacich et al., 2018; Muturi et al., 2018; Telang et al., 2018; Duguma et al., 2019). Sequencing of the 16S rRNA or18S rRNA hypervariable regions is used as a culture-independent approach to study mosquito microbiota composition (Pidiyar et al., 2004; Belda et al., 2017).

The mosquito gut microbiota is dominated by Gram-negative bacteria. A previous study identified 98 bacteria genera in anophelines, *Pseudomonas*, *Aeromonas*, *Asaia*, *Comamonas*, *Elizabethkingia*, *Enterobacter*, *Klebsiella*, *Pantoea*, and *Serratia* being the most common ones (Gendrin and Christophides, 2013). Similarly, Gram-negative bacteria are also dominant in *Aedes* spp. (Scolari et al., 2019).

However, unlike for the prokaryotic bacteria, the abundant 18S rRNA of the mosquito host strongly interferes with the definition of the eukaryotic microbiota composition via 18S rRNA gene sequencing. Thus, the mosquito eukaryotic microbiota remains poorly studied. Belda designed V4-region peptide-nucleic acid (PNA) oligonucleotide blockers to reduce by more than 80% mosquito 18S rRNA background for the detection of eukaryotic microbes (Belda et al., 2017). Most eukaryotic microbiota identified from mosquitoes belong to single cell eukaryotic phyla, such as *Candida*, *Pichia* with some *Penicillium* also being identified (Jupatanakul et al., 2014; Romoli and Gendrin, 2018; Thongsripong et al., 2018).

Bacteria colonize different mosquito organs, mainly midgut and rarely salivary glands, ovaries and male accessory glands (Tchioffo et al., 2015; Muturi et al., 2018). Most studies have focused on midgut microbiota. Mosquito salivary gland, ovaries and hemolymph are also important for virus or parasite replication and transmission. The adult mosquito midgut and ovary share some dominant bacteria classes, while other bacteria are only found in specific tissues or development stages (Tchioffo et al., 2015). Ovary bacteria can be vertically transmitted. *Wolbachia* is an intracellular bacterium that infects not only somatic tissue cells, but importantly also stably infects the germ cells of the ovary leading to vertical transmission (Hughes et al., 2014; Fraser et al., 2017; Jiggins, 2017). *Asaia*, an extracellular bacterium, can colonize the ovary of *Anopheles* mosquitoes and be vertically transmitted (Favia et al., 2007; Damiani et al., 2010). *Serratia* AS1, also an extracellular bacterium, was originally isolated from *Anopheles* ovaries, stably colonizes ovaries, and is transmitted vertically from female to progeny (Wang et al., 2017). Interestingly, *Serratia* AS1 also colonizes the accessory glands of male *Anopheles* mosquitoes, leading to sexually transmission (Wang et al., 2017).

## Impact of Microbiota on Mosquito Physiology and Pathogen Transmission

Mosquito microbiota play critical roles in many mosquito biology processes, including nutrition, digestion, mating and sexual reproduction, development, immune response functions, and refractoriness to pathogens (Douas, 2011).

## Impact of Microbiota on Mosquito Nutrition, Reproduction and Development

Dong et al. (2009) compared transcriptome between septic and aseptic adult female mosquitoes that had been fed different diets and found that some genes involved in digestion and metabolic processes such as glycolysis, gluconeogenesis and sugar transport, are stimulated by the presence of the microbiota. In *Aedes aegypti*, midgut microbiota, especially *Enterobacter* sp. and *Serratia* sp. isolates possess hemolytic activity that can lead to red blood cell (RBC) lysis and hemoglobin release (Gaio Ade et al., 2011). In *A. aegypti*, antibiotics treatment of female mosquitoes decreased the lysis of RBCs and egg production (Gaio Ade et al., 2011). However, egg production is not supported by every bacterium. Individual bacteria genera were used to populate adult mosquitoes emerged from gnotobiotic larvae. Five bacteria (*Aquitalea*, *Sphingobacterium, Chryseobacterium*, *Paenibacillus*, *and Comamonas*) were tested which supported egg production in *A. aegypti*, while in *A. atropalpus* only *Comamonas* supported egg production (Coon et al., 2016).

Mosquito microbiota can affect mosquito development. In *Anopheles*, a higher load of bacteria in the food diet sped larva growth and development (Linenberg et al., 2016). In *A. gambiae*, larvae carrying *Asaia* developed faster as it took 2 days less to reach the pupal stage than no*-Asaia* larvae (Mitraka et al., 2013). In *A. aegypti*, larval gut bacteria are crucial for growth and molting (Coon et al., 2017). Axenic larvae which are produced by surface sterilizing eggs, don’t molt and die as first instars; some species of bacteria which include *Escherichia coli* can colonize the midgut of axenic larvae and rescue larvae growth, while dead bacteria do not (Coon et al., 2014; Thongsripong et al., 2018). Larva gut microbiota consume oxygen and mediate hypoxia in the midgut. The hypoxia signal activates hypoxia-inducible transcription factors (HIFs) which activate several processes essential for larval growth, such as the insulin/insulin growth factor and mitogen activated kinases pathway (MAPK) (Vogel et al., 2017; Valzania et al., 2018). However, another study found that live bacteria are not required for *A. aegypti* larvae and adult development (Correa et al., 2018). In this study, a mixture of liver powder, yeast extract and heat-killed bacteria rescued axenic *A. aegypti* growth from larvae to adults. This result implies that a diet with the appropriate concentration of nutrients but not containing live bacteria appears to be sufficient to rescue larval development. In *Drosophila*, larval microbiota is essential for scavenging amino acids (Yamada et al., 2015). So, these studies suggest that larval gut microbiota may provide some essential nutrition (such as amino acids and proteins) which rescue axenic larvae growth and molting.

## Impact of Microbiota on Mosquito Refractoriness to Pathogens

Gut bacteria can influence the outcome of pathogen infections. Mosquito midgut microbiota induces peritrophic matrix formation and stimulate basal immune activity that protects the mosquito from pathogen infection (Barletta et al., 2017; Rodgers et al., 2017; Song et al., 2018; Yordanova et al., 2018). However, the effect of mosquito gut bacteria on parasite infection is complicated. A previous study showed that different strains of the genus *Serratia* can induce different outcomes on *Plasmodium* infections (Bando et al., 2013). Interestingly, a recent study reported that a *Serratia marcescens* strain isolated from a lab-adapted *A. aegypti* mosquito strain facilitates arboviral infection (Wu et al., 2019). Gloria-Soria studied more than 2,000 *A. aegypti* from 63 populations in 27 countries and did not find any natural infection by *Wolbachia* in *A. aegypti* (Gloria-Soria et al., 2018). *Wolbachia* has been applied to control arboviruses spread in *A. aegypti* mosquitoes. Moreira reported for the first time that *Wolbachia* infection reduces the ability of dengue and Chikungunya virus (CHIKV) to infect *A. aegypti* (Moreira et al., 2009). More recently *Wolbachia* was shown to also be a strong inhibitor of *A. aegypti* Zika virus infection (Dutra et al., 2016). Infection by the wMel strain of *Wolbachia* also can significantly reduce CHIKV and Yellow Fever virus (YFV) infection and dissemination rate (van den Hurk et al., 2012). However, a *Wolbachia* strain was reported to enhance vertical densovirus transmission by *Culex pipiens* (Altinli et al., 2018; King et al., 2018).

## Using Microbiota for Mosquito Population Reduction

Chemical insecticides have long been used for mosquito population control. However, a major problem is the development of insecticide resistance. Also, insecticides may have adverse effects, such as non-target killing and environmental disturbance. In contrast, use of the mosquito microbiota for population control minimizes the problem of resistance and show minimal negative effects to the environment. The best studied bacteria belong to the *Wolbachia* genus. Intracellular bacteria *Wolbachia* can infect approximately 2/3 of insect species. *Wolbachia* can vertical spread through the female germline to regulate insect reproduction. Cytoplasmic incompatibility (CI) is the main feature caused by *Wolbachia* in insects. when the uninfected females mate with *Wolbachia*-infected males, and lay eggs which cannot develop to larvae; however, if both of female and male parents are infected, embryos develop normally (Jiggins, 2017). Mosquito population reduction is achieved by releasing *Wolbachia*-infected male mosquitoes in the field. The understanding of the molecular bases for CI has long been an enigma. Recent studies showed that the *Wolbachia* deubiquitylating (DUB) enzymes cidA and cidB contribute to CI of mosquito zygotes (Beckmann et al., 2017). *Wolbachia pipientis* Type IV Effector WD0830 also plays an important role in CI (Sheehan et al., 2016). The *Wolbachia* genome encodes more than 20 ankyrin-repeat proteins, which may contribute to mosquito male offspring killing. Moreover, infection with some *Wolbachia* strains can shorten *A. aegypti* life-span (McMeniman et al., 2009). Harumoto and Lemaitre (2018) identified a toxin produced by the endosymbiont *Spiroplasma poulsonii* that selectively kills male *Drosophila* offspring. Recently, Zheng released *Wolbachia* infected *Aedes albopictus* to reduce mosquito population by offspring CI, and successfully reduce mosquito 88.7–96.6% biting in two isolated riverine islands in Guangzhou, China (Zheng et al., 2019).

## Exploitation of Microbiota to Combat Mosquito-Borne Diseases

Mosquito microbiota shows much potential to combat mosquito-borne diseases by rendering mosquito refractory to arthropod-borne human pathogens. For this purpose, the ideal microbe should have the following characteristics: easy genetic manipulation, efficient colonization of mosquitoes, be able to spread into mosquito populations (vertical and horizontal transmission), and effectively inhibit pathogen development in mosquitoes (Wang and Jacobs-Lorena, 2013; Wang et al., 2017).

The use of symbiotic bacteria to reduce the mosquito vectorial competence has gained increasing interest as an alternative approach toward disease control. This is based on two facts: (1) in initial stages of infection, the commensal microbiota and mosquito-borne pathogens share the same midgut compartment; (2) midgut microbiota proliferate dramatically after a mosquito blood meal, resulting in a corresponding increase of effector molecules secreted by the bacteria (Wang and Jacobs-Lorena, 2013).

Several reports have shown that the midgut microbiota can affect the infection of malaria parasite in its host mosquitoes (Pumpuni et al., 1993; Gendrin and Christophides, 2013; Wang et al., 2017). Some mosquito gut bacteria including *S. marcescens*, *Acinetobacter* sp. inhibit malaria parasite infection in mosquitoes (Cirimotich et al., 2011; Wang et al., 2017). However, mechanisms by which specific gut bacteria negatively impact malaria parasite development in the mosquito is largely unknown.

To exploit gut symbionts in the control of vector-borne disease transmission, genetic engineering has been used to modify certain symbiotic bacteria to produce anti-pathogen effector molecules (paratransgenesis) without affecting the fitness of the host vectors. In 1997, *Rhodnius prolixus* engineered with a gene encoding cecropin A, a peptide lethal to the parasite *Trypanosoma cruzi*, was introduced into the *R. prolixus* vector to control transmission of *T. cruzi* (Durvasula et al., 1997). The mosquito symbiotic bacterium *Pantoea agglomerans* was engineered to express anti-malaria effectors to interfere with malaria parasite development in mosquitoes (Wang et al., 2012). Recently, a new bacterium strain (AS1) of the genus *Serratia* isolated from the *Anopheles* ovary, was shown to stably colonize the mosquito midgut and reproductive organs. *Serratia* AS1 is transmitted vertically from the female to offspring and horizontally from male to female during mating, and spreads rapidly into mosquito populations. Moreover, *Serratia* AS1 can be engineered to express anti-malaria genes and mosquitoes that carry these bacteria are substantially refractory to the human malaria parasite *Plasmodium falciparum*. Thus, *Serratia* AS1 provides a powerful tool for driving mosquito refractoriness to *Plasmodium* infection (Wang et al., 2017). Another symbiotic bacterium *Asaia* can also colonize the mosquito midgut and reproductive organs (Favia et al., 2007). Recently, *Asaia* was also modified to express anti-malaria effectors and the engineered strains inhibit the development of malaria parasite (Shane et al., 2018). Reveillaud reported that *Wolbachia* from four wild *Culex pipiens* mosquitoes carry a plasmid (pWCP), opening the possibility of future paratransgenesis utilizing *Wolbachia* (Reveillaud et al., 2019).

## Concerns Relating to Potential Release of Genetically Modified Symbionts

While the feasibility of using paratransgenesis to contain the spread of malaria was demonstrated with laboratory experiments, translation of these findings to field application will need to overcome major regulatory barriers, as it involves the release of genetically modified (GM) organisms in nature. A basic requirement for the release of GM organisms is that benefits considerably outweigh the risks (Durvasula et al., 1997). Among issues that need to be considered is horizontal gene transfer (HGT). For mosquitoes, no study has been performed to evaluate potential transgene dispersion via HGT. For *R. prolixus*, a theoretical model was designed to predict HGT from a GM bacteria *Rhodococcus rhodnii* to a closely related bacterium, *Gordona rubropertinctus*, and predicted HGT frequency is less than 1.14 × 10^–16^ per 100,000 bacterial generations (Matthews et al., 2011).

## Concluding Remarks

The mosquito microbiota is acquired from the environment, and its composition is highly dynamic, varying depending on species, nutrition, development stage, and geography. Microbiota mostly colonize the midgut and rarely salivary glands and reproductive organs. The mosquito microbiota plays important roles in host nutrition, digestion, mating, sexual reproduction, development, immune functions and refractoriness to pathogens. Microbiota, GM or not, have been proposed for mosquito population control and combating mosquito-borne diseases. The introduction of GM symbionts engineered to produce anti-pathogen molecules into mosquitoes in the field shows much promise, but this can happen only after regulatory and public concerns are overcome.

A number of scientific questions remain to be addressed. First, many commensal bacteria may not always stop pathogen development in the mosquito. For example, *Serratia* inhibits malaria parasite infection of mosquitoes (Gonzalez-Ceron et al., 2003; Bando et al., 2013; Wang et al., 2017), while it promotes dengue virus infection of a culicine mosquito (Wu et al., 2019); A *Wolbachia* species reduces arbovirus infection of *A. aegypti* mosquitoes (Moreira et al., 2009; van den Hurk et al., 2012; Dutra et al., 2016) whereas another species enhances vertical densovirus transmission by *Culex pipiens* (Altinli et al., 2018; King et al., 2018). These apparently contradictory observations will only be clarified when the mechanisms underlying the observed effects are understood. Second, except for *Wolbachia*, no naturally occurring symbiont that can both inhibit pathogen infection and spread through mosquito populations has been identified. *Wolbachia* are effective in blocking viral transmission by *A. aegypti* but not to control transmission of the malaria parasite by anopheline mosquitoes. Identification of a naturally occurring bacterium that can inhibit *Plasmodium* transmission and spread through mosquito populations is an important future goal. Thirdly, the identification of effector proteins that specifically inhibit transmission of viruses such as dengue, zika, yellow fever and Chikungunya, and are harmless to the host vector, would allow implementation of disease control via paratransgenesis and mosquito transgenesis. Lastly, laboratory experimentation has demonstrated the high promise of paratransgenesis to fight mosquito-borne disease and a high priority should be given to address regulatory, ethical, and public acceptance issues.

## Author Contributions

All authors listed have made a substantial, direct and intellectual contribution to the work, and approved it for publication.

## Conflict of Interest

The authors declare that the research was conducted in the absence of any commercial or financial relationships that could be construed as a potential conflict of interest.
